# Non-targeted multimodal metabolomics data from ovine rumen fluid fractions

**DOI:** 10.1128/MRA.00392-23

**Published:** 2023-07-19

**Authors:** Nikola Palevich, Paul H. Maclean, Mingshu Cao

**Affiliations:** 1 AgResearch Ltd., Grasslands Research Centre, Palmerston North, New Zealand; Indiana University, Bloomington, Bloomington, Indiana, USA

**Keywords:** rumen, metabolomics, ovine, biofluid

## Abstract

From an animal health perspective, our understanding of the metabolites in rumen fluid across different host species is poorly understood. Here, we present a metabolomic data set generated using hydrophilic interaction liquid chromatography and semi-polar (C18) chromatography methods coupled to high-resolution mass spectrometry of fractionated ovine rumen samples.

## ANNOUNCEMENT

Ruminant livestock are an important component of feeding the growing human population while also being sources of global greenhouse gas emissions. The rumen is a strictly anaerobic environment enriched with a complex community of bacteria, protozoa, fungi, archaea, and bacteriophages. Rumen microbiota breakdown and convert plant proteins and polysaccharides from feed into energy sources but also result in methane formation that affects ruminant productivity. Metabolomics is a powerful and sensitive approach for investigating low-molecular-weight metabolite profiles present in rumen biofluids. It can be used to identify potential roles of metabolites in the rumen microbiome and provide understanding of host-level regulatory mechanisms associated with animal production. While rapid developments in genomics have accelerated our knowledge of rumen molecular biology (1), there has been less work focusing on the low-molecular-weight molecules that stem from rumen fermentation of feed and a complete absence of metabolomics studies on ovine rumen samples (2 - 8).

**Fig 1:**
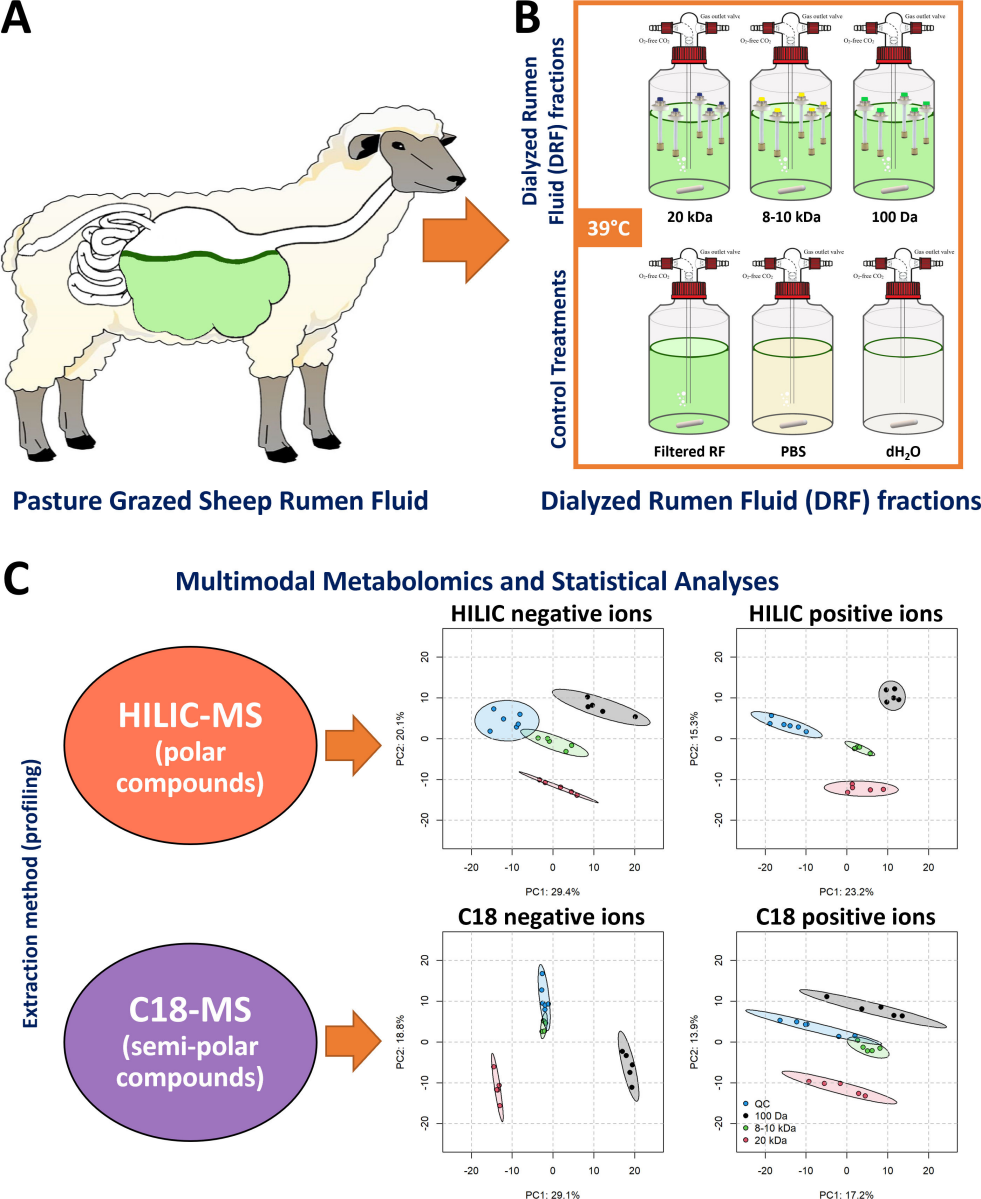
Overview of the experimental design, multimodal metabolomics workflow, and statistical analysis. (**A**) New Zealand pasture-fed sheep used for this study. (**B**) Dialyzed rumen fluid (DRF) fractions were obtained under anaerobic rumen conditions (39°C and CO_2_) using dialysis systems at three molecular weight cutoffs. (**C**) Schematic of a multimodal metabolomics workflow and statistical analyses used to process data integrated from multiple analytical approaches. Multigroup DRF fraction metabolic profiles across two metabolomics analyses. Principal component analysis (PCA) scores plot for the first two components of raw chromatographic peaks for C18 column negative ions (bottom left), C18 column positive ions (bottom right), HILIC column negative ions (top left), and HILIC column positive ions (top right). Each MWCO or DRF fraction was colored to represent 20 kDa (red), 8–10 kDa (green), 100 Da (black), and QC control (blue) samples. The QC comprised a pooled aliquot of the extract of all samples. The scatter plots shown represent plots with the two components having the greatest variations. Percentages on axes indicate the percentage of explained variance for the respective principal components. Ellipses are 95% confidence intervals of the scores of dialyzed rumen fractions. Observations that are similar will fall close to each other in clusters. One data point represents one biological replicate. HILIC, hydrophilic interaction liquid chromatography.

Whole rumen content samples were collected post-mortem and pooled from five sheep (Fig. 1A) grazing *ad libitum* on a ryegrass and clover pasture diet in Palmerston North, New Zealand (40°18′ S, 175°45′ E). A method was developed to acquire dialyzed rumen fluid (DRF) fractions that enrich for different sized components (Fig. 1B). DRF fractions based on three molecular weight cutoffs (MWCO) were obtained using Spectra-Por Float-A-Lyzer G2 dialysis systems with MWCOs of 20 kDa (Z726931, Sigma-Aldrich), 8–10 kDa (Z726605, Sigma-Aldrich), and 100 Da (Z727253, Sigma-Aldrich). Approximately 5 L of rumen contents was collected from each animal and filtered through four layers of cheesecloth (335 µm mesh) to account for the macro components of rumen fluid and transferred into Schott gas washing bottles fitted with Drechsel type head connections (GL 14, DURAN). To obtain each DRF fraction, replicates of each individual MWCO apparatus (*n* = 5 for each) were dialyzed against 10 mL of autoclaved phosphate buffered saline buffer overnight at 39°C in a water bath under anaerobic conditions obtained by insufflating a stream of O_2_-free CO_2_ inside the container with constant mixing. DRF samples were snap-frozen in liquid nitrogen, transferred to glass vials, and stored at −80°C until further use.

To comprehensively survey metabolites associated with DRF fractions, we used hydrophilic interaction liquid chromatography (HILIC) to separate polar compounds and C18 chromatography for separation of semi-polar compounds (Fig. 1C). Analyses were performed in both positive and negative electrospray ionization mode at the resolving power setting of 25,000 with a maximum trap fill time of 100 ms using the Xcalibur v4.3 software. The LC-MS raw data files were converted to mzXML files using MSConvert function of ProteoWizard v3 software (9). Quality control and peak deisotoping analysis were based on our previously published procedures (10). The details of extraction procedures, chromatographic gradients, and instrument settings have been previously described by Palevich et al. (11). For each metabolomic data set, principal component analysis (PCA) was performed on the log_10_ intensities for raw chromatographic peaks to assess similarity and separation of DRF fractions. The *mixomics* package v6.16.3 in R v4.1.1 was used to perform the PCA and generate plots.

Overall, according to the PCA scores plots (Fig. 1C), each of the three DRF fractions had considerably different profiles for both types of metabolomic analyses and regardless of ionization mode. The three DRF fractions separated completely either on the first or second component or a combination thereof within the 95% CI ellipse. This study highlights the potential of HILIC and C18 chromatography combined with non-targeted mass spectrometric methods to detect the polar and semi-polar metabolite species of the ruminal fluid metabolome. The presented untargeted metabolomics data provide a detailed snapshot of the ovine ruminal fluid metabolome that can be used as a reference for future studies of the rumen metabolome or as a comparator for other ruminant species.

## Data Availability

The data sets presented in this study and supporting the conclusions of this article have been made available in the MetaboLights database (MTBLS1717) online repository.
